# Wireless Module for Nondestructive Testing/Structural Health Monitoring Applications Based on Solitary Waves

**DOI:** 10.3390/s20113016

**Published:** 2020-05-26

**Authors:** Ritesh Misra, Hoda Jalali, Samuel J. Dickerson, Piervincenzo Rizzo

**Affiliations:** 1 Mixed-Signal Multi-Domain Systems Laboratory, Department of Electrical and Computer Engineering, University of Pittsburgh, 3700 O’Hara Street, 1206 Benedum Hall, Pittsburgh, PA 15261, USA; rim39@pitt.edu (R.M.); dickerson@pitt.edu (S.J.D.); 2 Laboratory for Nondestructive Evaluation and Structural Health Monitoring Studies, Department of Civil and Environmental Engineering, University of Pittsburgh, 3700 O’Hara Street, 729 Benedum Hall, Pittsburgh, PA 15261, USA; HOJ14@pitt.edu

**Keywords:** highly nonlinear solitary waves, nondestructive evaluation, wireless sensing, Bluetooth technology

## Abstract

In recent years, there has been an increasing interest in the use of highly nonlinear solitary waves (HNSWs) for nondestructive evaluation and structural health monitoring applications. HNSWs are mechanical waves that can form and travel in highly nonlinear systems, such as granular particles in Hertzian contact. The easiest setup consists of a built-in transducer in drypoint contact with the structure or material to be inspected/monitored. The transducer is made of a monoperiodic array of spherical particles that enables the excitation and detection of the solitary waves. The transducer is wired to a data acquisition system that controls the functionality of the transducer and stores the time series for post-processing. In this paper, the design and testing of a wireless unit that enables the remote control of a transducer without the need to connect it to sophisticated test equipment are presented. Comparative tests and analyses between the measurements obtained with the newly designed wireless unit and the conventional wired configuration are provided. The results are corroborated by an analytical model that predicts the dynamic interaction between solitary waves and materials with different modulus. The advantages and limitations of the proposed wireless platform are given along with some suggestions for future developments.

## 1. Introduction

Highly nonlinear solitary waves (HNSWs) are mechanical waves that can form and travel in highly nonlinear systems such as mono-periodic arrays of elastically interacting spherical particles, sometimes indicated as granular crystals [1,2,3,4,5,6,7,8,9,10,11,12,13,14,15,16,17,18]. One of the key features of HNSWs is that properties like duration, amplitude, and speed can be tuned without electronic equipment by simply adding static precompression on the array or varying the particles’ material and geometry. This tunability makes the use of HNSWs appealing in some engineering applications such as nondestructive testing (NDE), structural health monitoring (SHM) [19,20,21,22] or acoustics [23,24,25,26,27,28].

**Figure 1:**
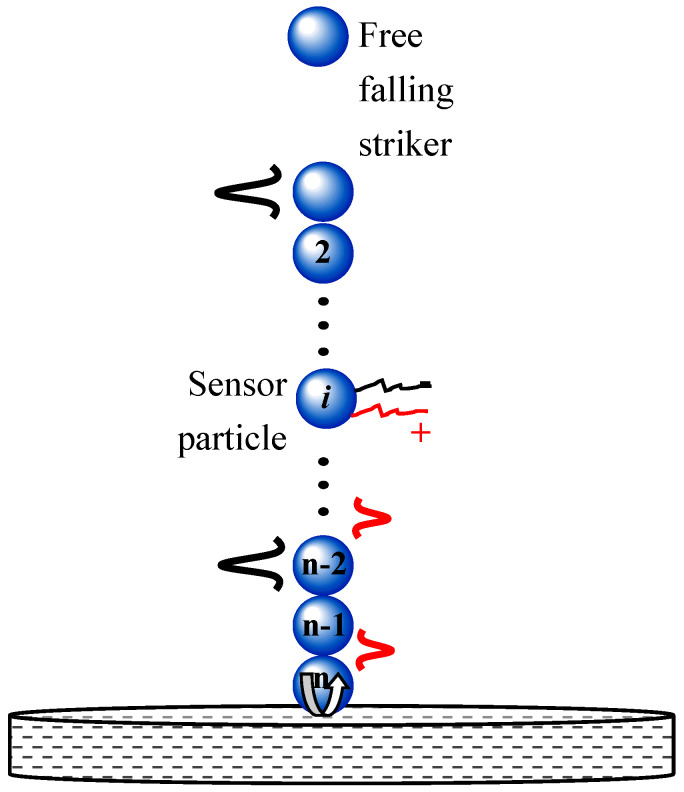
General scheme of nondestructive evaluation/structural health monitoring paradigm using highly nonlinear with different modulus. The advantages and limitations of the proposed wireless platform are given along with some suggestions for future developments.

The scheme of an HNSW-based NDE/SHM is shown in Figure 1. One end of an array of identical spheres is in dry contact with the structure to be inspected/monitored. The top particle of the chain is lifted and released to create a mechanical impact that triggers the formation of a solitary wave, hereinafter referred to as the incident solitary wave (ISW). When the mass of the striker is equal to the mass of the other particles composing the chain, a single pulse is generated. The ISW propagates through the array, is detected by a sensing system embedded in the chain, and then reaches the surface of the structure to be inspected/monitored. At the interface between the chain and the structure, most of the acoustic energy carried by the ISW is reflected back, giving rise to one or two reflected solitary waves, typically referred to as the primary and the secondary reflected solitary waves (PSW and SSW). These reflected waves are then detected by the same sensing system embedded in the array.

Many researchers proved that the amplitude and the arrival time of the reflected solitary waves depend on key properties of the adjacent structure/material [19,20,21,22,29,30,31,32,33,34,35,36,37,38]. For example, Yang et al. studied numerically, analytically, and experimentally the reflection of HSNWs at the interface of a large thin plate, and found that the amplitude and the arrival time of the reflected waves are affected by the plate thickness, the particles size, and the boundary conditions at a critical distance from the plate edges [39]. Kim et al. indicated experimentally and numerically that solitary waves can be used to detect delamination in carbon fiber reinforced polymer plates [31]. A numerical study on the interaction of HNSWs with composite beams showed that solitary waves are helpful to evaluate the directional elastic parameters of the composites [40]. Others reported that the method is effective at detecting subsurface voids [38], assessing the quality of adhesive joints [22,30], composites [31,34,40,41], orthopedic and dental implants [32,42], and at measuring internal pressure [43,44,45,46] and axial stress [47,48].

In all the studies cited above, the array of particles is coaxially wired to electronic equipment that controlled the striker and digitized the time-waveforms for post-processing analysis. Although this is acceptable for periodic inspections, i.e., NDE applications, it may be detrimental when continuous monitoring, i.e., SHM protocol, is preferred or necessary. SHM systems, however, can become expensive in large structures or high-dense sensor systems due in part to cabling networks. SHM using wireless sensors can overcome the limitations of traditional wired methods with many attractive advantages such as wireless communication, onboard computing, battery power, ease of installation, and so on. Platforms for SHM containing wireless smart sensing (WSS) have been developed during the past years. WSSs are devices that have a sensor, microprocessor, radio frequency transceiver, memory, and power source integrated into one small size unit and are characterized by their capabilities of sensing, computation, data transmission, and storage, all achieved by a single device. WSS represents an attractive alternative to their wired counterparts because of the lower cost achieved by removing the need for cables (including cost labor reduction), and by the widespread production of micro-electro-mechanical sensors. The wireless communication capability allows flexible network topology and hence enables a decentralized monitoring scheme, which adds robustness to the SHM system compared with the centralized approach in wired systems [49]. For these reasons, in the last two decades there has been a great deal of research and development of wireless sensors for SHM applications that were summarized in a few excellent reviews [50,51,52,53]. However, as the NDE/SHM method based on HNSW has been developed only recently, there is no wireless sensing technology available in support of nonlinear solitary wave propagation and detection.

To fill this gap, a wireless sensor system to support the generation and detection of HNSWs for NDE or SHM applications was designed, assembled, and tested. The system is not a sensor per se, but rather an autonomous data acquisition node to which a traditional HNSW transducer, designed and developed in our lab, can be attached. The new device can be best viewed as a platform in which mobile computing and wireless communication elements converge with the transducer. This capability is particularly advantageous in the context of SHM, in which several HNSW transducers can be deployed on the structure of interest to perform structure interrogation and remote communication to a remote repository. Other advantages of this newly designed system are cost reduction in the installation phase, autonomous data processing, denser sensing capability in large structural systems, just to mention a few.

The paper is organized as follows: Section 2 provides an overview of the designed wireless platform and is divided into sub-sections that describe the single components. Section 3 presents the experimental results in which the wireless platform is compared to the performance of conventional sensing with the use of a data acquisition system. Section 4 supplements the experimental finding with the outcomes of a numerical analysis that models the dynamic interaction of the solitary waves with different materials. Lastly, Section 5 provides some concluding analyses and remarks about the study presented here.

## 2. Wireless HNSW Sensor System Overview

**Figure 2:**
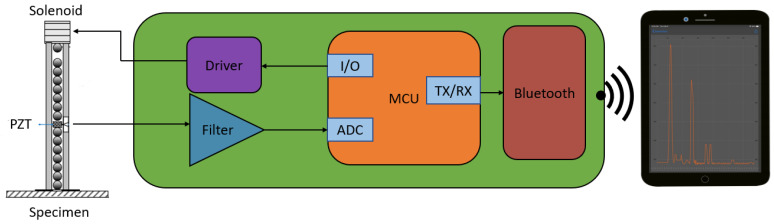
Schematics of the overall HNSW wireless sensor node. The “specimen” in the figure representsany material/structure to be monitored using the solitary waves.

The new wireless sensor network is comprised by three components, the HNSW transducer, a custom printed circuit board (PCB), and a mobile computing device. A schematic of the overall platform is shown in Figure 2.

### 2.1. HNSW Transducer: Conventional Design

The HNSW transducer, hereinafter simply referred to as the *transducer*, contains eight 19.05 mm spheres, a commercial electromagnet (Uxcell 12 V DC) able to lift and release the striker, a sensor, and a frame. All the particles except the striker were made of a non-ferromagnetic material. The current flowing through the solenoid generates a magnetic field strong enough to lift the first particle of the array, whereas flow interruption causes the striker to impact the chain and generate the ISW. The sensing system consists of a lead zirconate titanate (Pb[Zr_x_Ti_1-x_]O_3_) wafer transducer (PZT), embedded between two 6.05 mm thick, 19.05 mm diameter disks. Kapton tape insulated the PZT from the metal. In the conventional (wired) configuration, the transducer is connected to and driven by a National Instruments PXI running in LabVIEW using a graphical user interface created ad hoc for the experiments. The hardware generates the signal to open/close the electrical current to feed the solenoid, while the user interface allows for the selection of the repetition rate of the striker (by controlling the output signal), the number of measurements, the sampling frequency of the digitized waveforms, and the storage of the waveforms for post-processing analysis. The *transducers* used in this study were also successfully used elsewhere [19,43,44,45,54] and were, therefore, the starting point to develop the proposed wireless platform. However, it is emphasized here that the proposed platform can be adopted and adapted to a chain of any size, length, and particle materials.

### 2.2. Circuit Board

In the proposed wireless sensor node, the role of the data acquisition system used in the conventional configuration is replaced by a PCB, which is the centerpiece of the schematics of Figure 2. The PCB includes the driver to provide the current for the solenoid, a filter to remove white noise and provide anti-aliasing, and a protocol for the remote communication to and from mobile devices.

**Figure 3:**
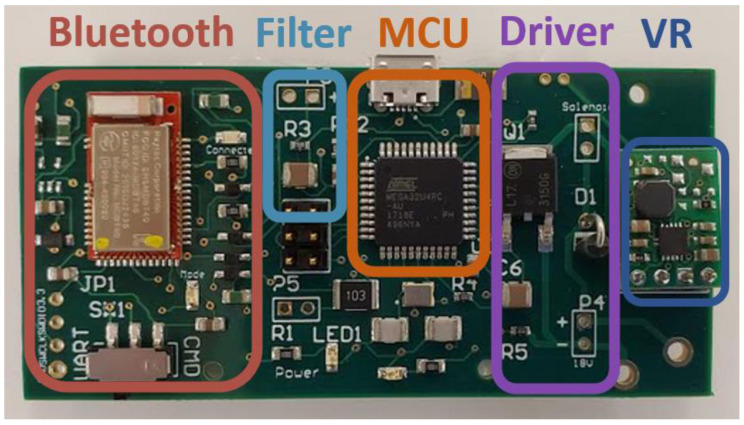
Photo of the final printed circuit board (PCB), which includes a Bluetooth transceiver, filter, microcontroller (MCU), transducer driver, and a voltage regulator (VR). The board is 76.2 mm wide and 36.8 mm high.

Figure 3 shows a photo of the PCB with color-coded sections corresponding to the individual components. The driver allows the microcontroller (MCU) to deliver the DC current to the solenoid with an input/output (I/O) pin on-demand (GPIO). Although the term “GPIO” suggests that both an output and an input are necessary to interface with the driver, the latter is designed such that the movement of the striker onto the array can be controlled through a single digital output. The waves, sensed by the PZT, are filtered by an analog filter and subsequently sampled by the analog to digital converter (ADC) within the MCU. The MCU then sends the data samples to an integrated circuit (IC) enabled for Bluetooth Low Energy (BLE) communication using the Universal Asynchronous Receiver/Transmitter (UART) protocol. The protocol allows for the transmission of the data to any mobile device capable of BLE communication.

The final PCB (76.2 × 36.8 mm^2^) was designed to optimize space and therefore enhance portability. The MCU was an ATMega32u4 with 32 kB of flash memory for storing embedded programs, 2 kB of SRAM for storing measurement data, all of the peripherals required to induce and measure the signal, and libraries that allowed for easy communication with the Bluefruit LE module. The MCU has also its own USB controller, making local data collection possible without adding an IC to do FTDI to UART conversion. Overall, the created design fares better than the conventional design because the new system is more compact, is dedicated to the specific application with HNSWs, and can be driven by smart devices such as tablets or even smartphones.

### 2.3. Actuation: Wireless Configuration

In the wired configuration, the DC current used to drive the electromagnet is delivered by a power supply external to or embedded to a data acquisition system. In the proposed wireless node, the DC current is provided by the PCB. Following the protocol implemented in the PXI in previous studies [19,43,44,45,54], the solenoid is energized for 250 ms, an interval sufficiently long to lift the striker until it touches the electromagnet before falling freely onto the array. The energy necessary to deliver the current necessary to operate the electromagnet is significant with respect to the other electronic components and is directly proportional to the weight and the falling height of the striker. To supply the necessary energy, a custom power source for the solenoid and driver circuit was chosen to allow the control of the striker while maintaining portability. A 14.8 V rechargeable LiPo battery with a maximum discharge rate of 47 A and a capacity of 1050 mAh was chosen. For the 250 ms interval, 675 mA is consumed. In future, the duration and/or the energy consumption can be reduced by shortening the falling height of the striker, by making the striker lighter (in order to be able to use smaller solenoids), or by minimizing the friction between the striker and the inner wall of the guide.

Under this design, the battery can continuously drive the striker for 93 minutes, which means that the battery can theoretically power 22,390 = (93 × 60 × 4) impacts. This value was obtained by multiplying the capacity of the battery by the inverse of the current consumption. The number of strikes can be also increased using a battery with higher capacity within the limit of size and weight constraints necessary to make the whole node practical. In the present study, the 1050 mAh battery was chosen because it was sufficient to complete a full round of experiments (see Section 3).

**Figure 4:**
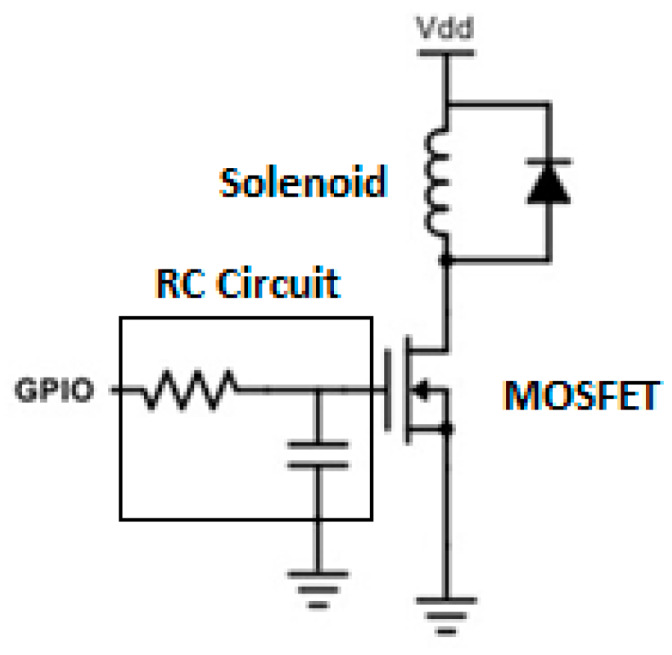
Schematic of the control circuit used to drive the direct current through the solenoid. *V_dd_* represents the 14.8 V power source while the coil directly below *V_dd_* represents the electromagnet used to lift the topmost ball.

Figure 4 shows the control circuit for the actuation. A 1N4003 diode was added in parallel to the solenoid; this flyback diode prevents voltage spike that results from turning off the solenoid from damaging the metal–oxide–semiconductor field-effect transistor (MOSFET), which would, in turn, shorten the life of the overall system [55]. The MOSFET itself acts as an open circuit if the GPIO pin is off, and acts as a closed circuit if the GPIO pin is on, allowing for the control of the current through the solenoid via software. The MOSFET NTD3055-150 from ON Semiconductor was chosen. According to the manufacturer, this specific MOSFET is designed for low voltage, high-speed switching applications in power supplies, converters and power motor controls and bridge circuits, and dissipates 28.8 W continuously, which provides a threefold factor of safety compared to the 9.45W (14 V × 0.675 A) required to lift the striker. The calculation for the required wattage was based on experiments done with a power supply that justified the choice of a 14.8 V battery in the first place. An RC circuit at the gate of the transistor provides a slight delay between turning the GPIO pin off in software and the moment at which the magnetic ball on top of the chain drops. The specific resistor and capacitor values used are not critical, what is most important is that the time constant of the RC circuit is three times larger than the minimum delay that the MCU can produce. This prevents the worst-case scenario of the MCU sampling the ADC after the incident waves passed the sensor disk. The delay that the RC circuit introduces safeguards against mechanical adjustments to the transducer that can reduce the amount of time it takes for the striker to fall. The RC circuit consisted of a 10 kΩ resistor and a 33 nF capacitor, resulting in a time constant of 333 µs.

### 2.4. Filter Design

**Figure 5:**
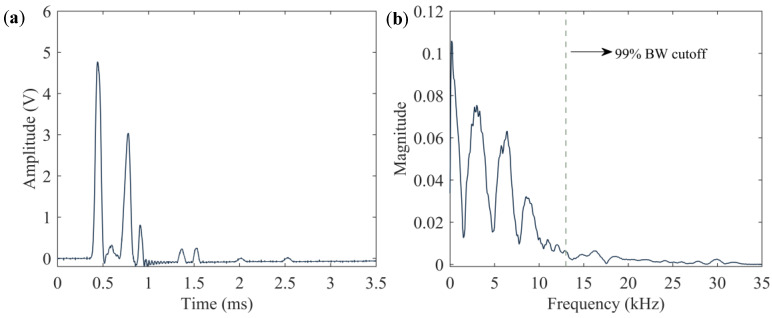
(**a**) Time waveform and (**b**) corresponding Fast-Fourier transform (FFT) measured during a control test to design a low-pass filter.

A passive low-pass filter was added to remove white noise and provide anti-aliasing. The cutoff frequency was determined by examining the frequency spectrum of solitary waves recorded conventionally at a sampling rate of 2 MHz by placing a *transducer* above a 12.7 mm thick steel plate. Figure 5 shows one of the time waveforms and the corresponding Fourier transform associated with this control test. The 99% bandwidth of this signal (the frequency range across which 99% of the power in the signal is contained) is 13.93 kHz. Our initial approach was to place the cutoff frequency somewhere between 50 and 100 kHz so as to eliminate white noise and also have at least a 4x factor of safety from losing any important information. This would also have served to provide anti-aliasing. However, setting the cutoff frequency to a point within that range would introduce a significant amount of phase lag in the relevant frequencies, which could distort the measurements of the arrival time of the pulses of interest. The phase response of a filter is the radiant phase shift added to the phase of each sinusoidal component of the input signal, and the term ”phase lag” is used when these phase shifts cause delays in time. In the case that the primary wave and secondary wave have the exact same frequency content, both waves would experience the same amount of delay. However, if the reflected waves are different in terms of the frequency content, a narrow low-pass filter may cause artifacts. Based on the above, a cutoff frequency of 2.4 MHz was chosen in order to minimize the phase lag across the frequencies with relevant information.

**Figure 6:**
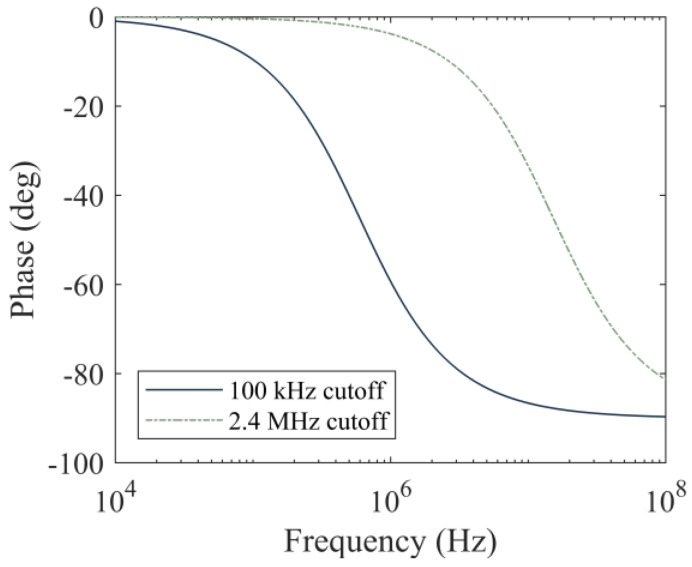
Phase lag as a function of frequency, induced by low pass filters with cutoffs at 100 kHz and 2.4 MHz.

Figure 6 compares two cutoff frequencies, namely 100 kHz and 2.4 MHz, and their effects on the phase response of the low pass filter. The phase lag in the 10–100 kHz range associated with the 2.4 MHz cutoff is essentially flat. At 50 kHz, the phase lag is 0.2 degrees which corresponds to a 0.01 µs delay, well below the sampling period typically used in HNSW tests. However, for the cutoff at 100 kHz, the phase lag at 50 kHz is equal to 4.8 degrees, which yields a 0.26 µs delay. A delay of 0.26 µs is negligible at a sampling period of 13.3 µs, which is the sampling period of the current design. However, as discussed within Section 6, we plan on increasing the sampling frequency in future iterations of this circuit board to at least 1 MHz. In that context, the usage of a lower cutoff frequency could distort the HNSW measurements. The components of the filter’s final design have values equal to 2 Ω and 33 nF, resulting in a cutoff frequency of 2.411 MHz. It is noted here that the design of the filter can be modified based upon the specific application of the transducers as the characteristics of the particles in the array and/or the properties of the structure to be monitored, modify the duration of the incident and reflected waves.

### 2.5. Wireless Communication

Bluetooth technology was chosen as the communication protocol between the newly proposed wireless device and laptops, tablets, or phones. For short-distance communication, Bluetooth technology is appealing owing to its compatibility with the majority of smartphones, tablets, and laptops. Additionally, Bluetooth communication does not rely on any external network. The Bluetooth LE UART module we used relies on the general-purpose, ultra-low power System-on-Chip nrF51822 to provide wireless communication with any BLE-compatible device. The term “System-on-Chip” means that the nrF51822 is a complete computer system within a single chip that can act independently from the MCU. This could be essential for improvements to future iterations of the PCB as discussed in the Discussion and Conclusions section. The nrF51822 has the ability to choose between UART and SPI communication with external devices, and sleep modes for power preservation. According to the manufacturer, the range of the module is approximately 60 m in an indoor environment, assuming a lack of obstacles. The module was flashed with the firmware provided by the manufacturer [56].

### 2.6. Mobile Application

**Figure 7:**
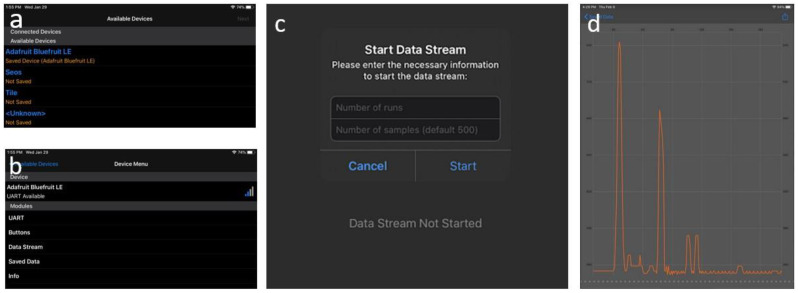
Screenshots of the custom application for mobile devices such as tablets and phones. (**a**) Panel to select the appropriate module from a list of devices. (**b**) Panel to select a data stream from a device menu. (**c**) Panel to enable the user to select the desired number of samples and runs. (**d**) Display of a single measurement from a sheet of aluminum plotted on the mobile device.

An app (Figure 7) was designed to communicate with the *transducer* via the PCB from a smart device. The app was adapted from a general app framework provided by the manufacturer [57]. The custom adaptation added a data streaming mode capable of compartmenting the data it received from different runs into separate graphs. These plots can also be exported as data files for further processing. The data streaming was designed to work with the messaging protocol programmed into the MCU, as discussed later in Section 2.7. First, the app provides a list of Bluetooth devices within the vicinity. After the user selects the appropriate device (Figure 7a), the “Data Stream” menu option allows the user to remotely drive the striker and collect data from the embedded sensor disk (Figure 7b). As shown in Figure 7c, selecting “Data Stream” prompts the user to select the number of strikes and the length (data points) of the signal. Once the PCB receives the command, it actuates the *transducer*, collects samples of the time waveform from the ADC, sends the data to the mobile device, and iterates the process as many times as the number of strikes chosen by the user. During the process, the waveforms are displayed in real-time on the smart device (Figure 7d). The app and the PCB together make a self-contained system that only requires a basic knowledge of smart mobile devices to operate.

### 2.7. Implementation

The ADC within the AtMega32u4 was used to digitize the signals detected by the embedded sensor disk. The ADC clock was set equal to 1 MHz, as setting the clock to a higher frequency would reduce the resolution for this particular device. A single conversion takes 13 clock cycles, and the clock frequency was set to 16 MHz, so the highest sampling frequency we could theoretically achieve was 1 (MHz)/13 = 77 kHz. The ADC uses a sample-hold capacitor, which is first charged by the signal and then closed-off from the input signal so that the voltage of the signal at that time can be indirectly read through the voltage on the capacitor at that moment. A 5 V power supply for the ATMega32u4 and the Bluetooth module was generated with a 3.7 V single-cell LiPo and a Pololu 5 V Step-Up Voltage Regulator U1V11F5. The U1V11F5 can handle input voltages in a range of 1 to 5.5 V, so it is robust to small voltage drops caused by the discharging of the single-cell LiPo. The PCB follows a protocol for collecting data and sending it to a mobile app. After the first time the PCB is turned on, it waits for a mobile device to connect to it. After a device has connected, the PCB turns the solenoid on and off again, starts a timer, and then collects ADC samples until the ADC reading passes a certain threshold. This allows it to learn the timing between it dropping the ball and observing a HNSW. It then allows the app to send it the desired number of samples and runs and then executes the appropriate number of runs while recording the desired number of samples in time for each run.

**Figure 8:**
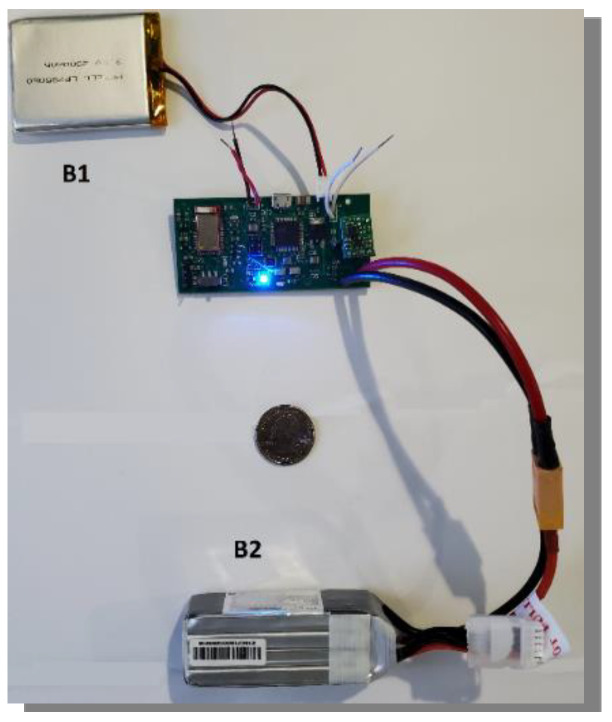
Photo of the final PCB with two batteries, B1 and B2. The 3.7 V battery (B1) powers the microcontroller and Bluetooth module. The 14.8 V battery (B2) powers the solenoid.

Figure 8 shows the final prototype used in the experiments presented in the next section. The PCB is connected to both batteries it needs for all of its functionalities and is in a state where it could be connected to any Bluetooth-compatible mobile device.

## 3. Experimental Setup and Procedure

**Figure 9:**
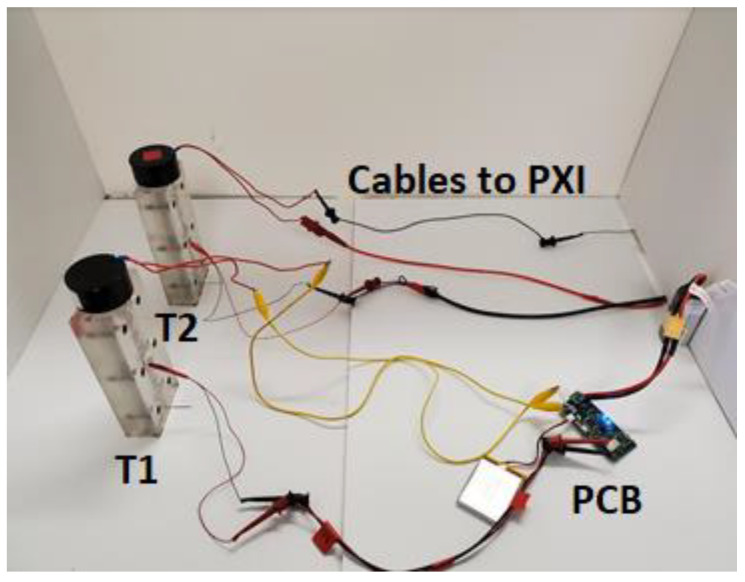
Photo of the two identical *transducers* used in this study. *Transducer* T1 was wired to the PCB. *Transducer* T2 was wired to a National Instruments PXI.

To compare the performance of the proposed wireless module to a conventional configuration, two identical *transducers* like those illustrated in Section 2.1 were used. Figure 9 shows part of the setup. *Transducer* T1 was wired to the PCB whereas *transducer* T2 was cabled to a National Instruments —PXI. The latter controlled an external DC power supply to energize the electromagnet.

Three sets of experiments were conducted. In the first two, both *transducers* were placed above a steel thick plate and then above a foam board. The latter was laying above an optical table. Then, one *transducer* only was placed above a granite block and connected to the wireless node first and then to the PXI. The steel specimen was 304.8 × 152.4 × 76.2 mm^3^, the foam board was 604.5 × 457.2 × 6.35 mm^3^, and the granite block was 152.4 × 152.4 × 304.8 mm^3^. For each experiment, 100 impacts were triggered in order to collect a statistically significant amount of data. For the steel plate, one transducer at the time was placed at the center of the 304.8 × 152.4 mm^2^ area. For the foam test, the two *transducers* were placed symmetrically, 101.6 mm away from the center of the board and 228.6 mm from the sides in order to secure identical boundary conditions. The three materials were chosen as representatives of different Young moduli. For the steel plate test, the sampling frequency was equal to 61 kHz and then raised to 75 kHz for the other two tests for both the wireless node and the PXI. Based on the discussion of Section 2.4, when the sampling frequency was 61 kHz, the filter used a 51 Ω resistor and a 330 nF capacitor, whereas when the sampling frequency was 75 kHz, a 2 Ω resistor and a 330 nF capacitor were used.

## 4. Experimental Results

### 4.1. Steel Plate: Control Test

**Figure 10:**
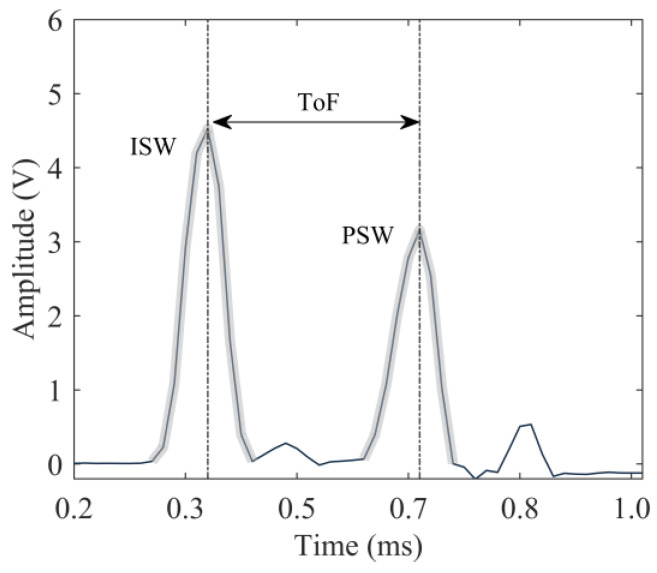
Steel plate test. Example of one of the hundred-time waveforms recorded during the experiment.

The first round of experiments was conducted on the thick steel plate and served as a control test to troubleshoot any possible problems associated with the wireless module. Figure 10 shows one of the time waveforms collected by *transducer* T1. The first peak is the ISW whereas the second peak around 550 µsec is the PSW. Both the ISW and the PSW were tailed by small humps. The origin of these small pulses is described in Section 5. It is anticipated here that these pulses are not detrimental to the successful implementation of NDE/SHM strategies with HNSWs. As conventionally done in HNSW-based NDE/SHM, the time-of-flight (ToF) of the PSW was calculated, as the difference between the arrival time of the PSW with respect to the ISW at the sensing disk. This wave feature is significant for NDE-based or SHM-based applications, as it has been demonstrated that the ToF is sensitive to the presence of damage and/or variation in local stiffness, etc.

**Figure 11:**
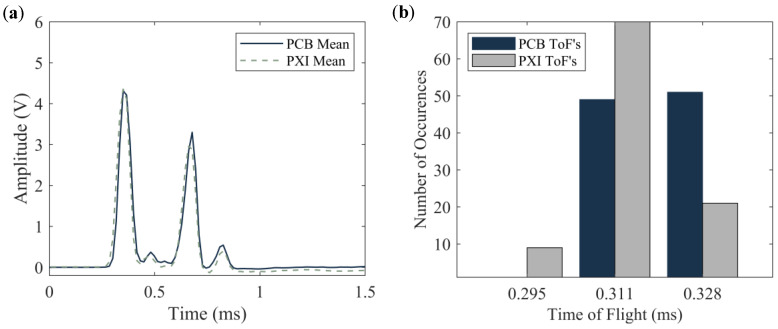
Steel plate test. (**a**) Time-series obtained by averaging the 100-time waveforms measured with the sensor disks. (**b**) Number of occurrences of a given ToF. For each color, the total number of occurrences is 100.

To compare the results from both the wired and the wireless systems, the time-series obtained by averaging the one hundred waveforms from both (wired and wireless) systems are displayed in Figure 11a. As the trigger for the two systems was different, the graphs in Figure 11a were shifted horizontally in order to overlap the arrival time of the ISW. Figure 11a proves that the two time-waveforms are remarkably similar to each other, in terms of pulse amplitude and corresponding ToF. The slight differences can be attributable to manual *transducer*-to-*transducer* differences associated with fabrication and assembly. To identify differences at each measurement, Figure 11b shows the number of occurrences for a given ToF for both setups. Three values, namely 295, 311, and 328 µs were measured. It is noted here that the width of each bar is equal to the sampling period, which, for the steel plate test, was equal to 16.4 µs.

### 4.2. Foam Specimen

**Figure 12:**
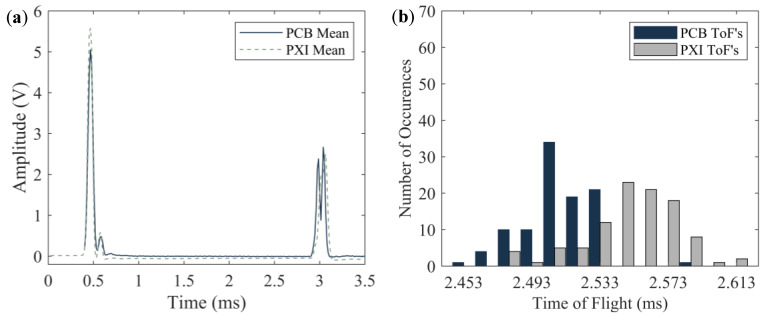
Foam board test. (**a**) time-series obtained by averaging the 100-time waveforms measured with the sensor disks. (**b**) Number of occurrences of a given ToF. For each color, the total number of occurrences is 100.

Similar to Figure 11 and Figure 12 presents the results associated with the foam board that was probed with both *transducers* acting sequentially. For this material, the amplitude of the ISW measured with the PXI is larger than the corresponding amplitude measured with the wireless platform. This trend confirms what seen in Figure 11a but for the latter case the difference was smaller. The origin of such difference is discussed in Section 4.3. Nonetheless, it is important to emphasize that for NDE/SHM applications, the trends of interests are those associated with variations from baseline data when material degradation of damage occurs. As such any *transducer* to *transducer* difference in the amplitude of the ISW is not detrimental to prove the main hypothesis of the present research, i.e., that the wireless platform can be used for HNSW measurements in lieu of sophisticated electronics. Another distinctive feature of the wireless platform is that it was able to detect a twin peak associated with the dynamic interaction of the waves with the foam. The origin of these twin-peaks, originally identified and detailed in [58] and elaborated further in [43], was found in several HNSW-based NDE related to soft materials including rubber [44]. For the sake of completeness, the dynamic interaction among the particles that induce the “twin-peaks” is discussed in Section 5.

Figure 12b shows that the number of occurrences of the ToF is more spread with respect to the steel test. This is attributable to the soft nature of the foam specimen. Additionally, the occurrences of the ToF measured with the wireless platform are skewed to the left, i.e., the PSW traveling along *transducer* T1 seems faster than in *transducer* T2. While the origin of such skew is not clear, and more insights could have been gained by inverting the terminals of the *transducers*, it is believed that what was observed is more related to the dynamic interaction of the waves with the foam rather than a variability associated with the *transducers*. In the future, the experiments will be repeated by connecting the terminals of the solenoid and the sensor disk to the PCB first and then to the PXI.

### 4.3. Granite Specimen

**Figure 13:**
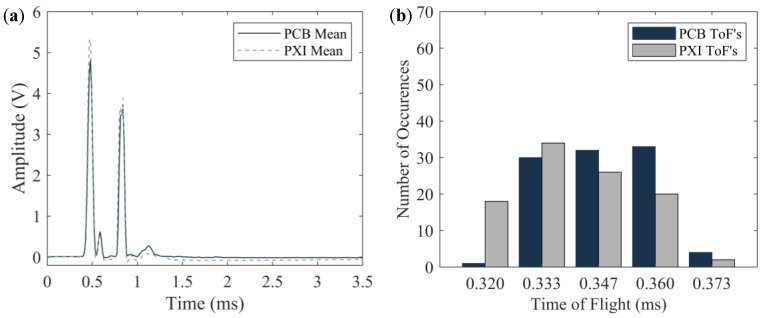
Granite test. (**a**) time-series obtained by averaging the 100-time waveforms measured with the sensor disks. (**b**) Number of occurrences of a given ToF. For each color, the total number of occurrences is 100.

To investigate the origin of the small discrepancies observed for the foam board, one transducer only was used above the granite block and connected to the PCB first and then to the PXI. The results are presented in Figure 13. The time-waveforms (Figure 13a) confirms the trend observed for the foam (Figure 12a). The amplitude of the ISW measured with the PXI is about 20% higher than the corresponding amplitude measured with the wireless node. As such, it can be concluded that differences in the impedance between the two devices result in differences in the amplitude of the detected incident wave. Figure 13b shows the number of occurrences associated with the granite test. The distribution of the ToF is in between the foam and the steel specimen. This confirms that the scattering of the results (i.e., the number of histograms plotted in each figure) is related to Young’s modulus of the material. Contrary to what is observed in Figure 12b or Figure 13b shows that the ToF measured with the PCB is higher than the ToF measured with the PXI.

## 5. Numerical Results

**Figure 14:**
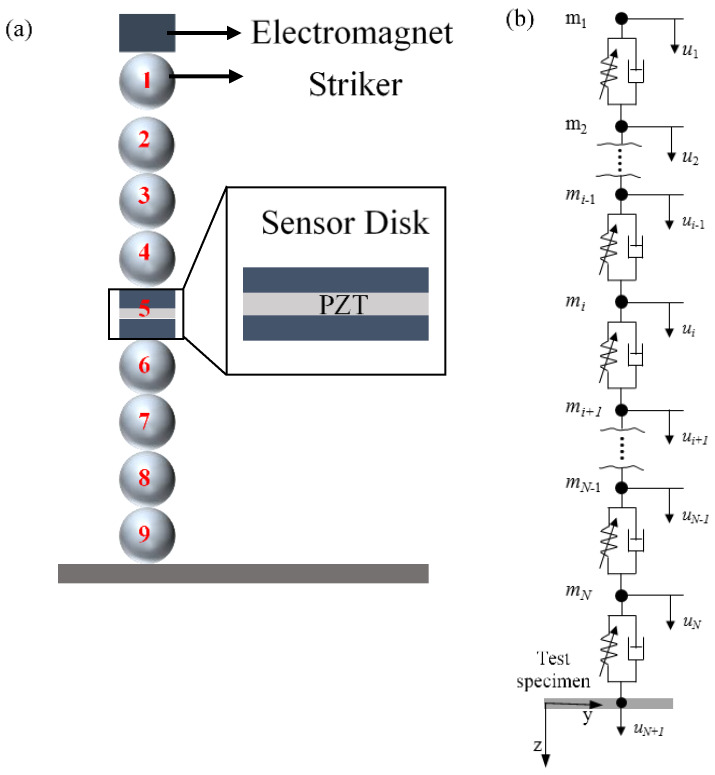
Modeling the interaction between the granular chain and the test specimen. (**a**) Schematics of the HNSW transducer with particle site number. (**b**) Schematic of the mono-periodic array as a series of point masses interacting via nonlinear Hertzian contact (In this figure, N = 9).

To interpret the experimental results presented in Section 4, an analytical study was performed about the dynamic interaction of solitary waves with the test specimens, using a model that simulates the propagation of solitary waves along the granular array using a series of point masses interacting via nonlinear Hertzian contact forces (Figure 14). Furthermore, the test specimen in contact with the granular chain was modeled as a linear elastic media using a finite element model. The two models were integrated at the interaction point between the granular chain and the test specimen based on the Hertzian contact law. Owing to the scope of this paper, the numerical formulation and the analytical equations used for the simulation are not presented here and interested readers are referred to [27,43,44,45,46,47,48,54] in order to gain more insight on the subject.

For the models, the properties listed in Table 1 were used for the three materials, namely steel, granite, and foam considered in this study.

**Figure 15:**
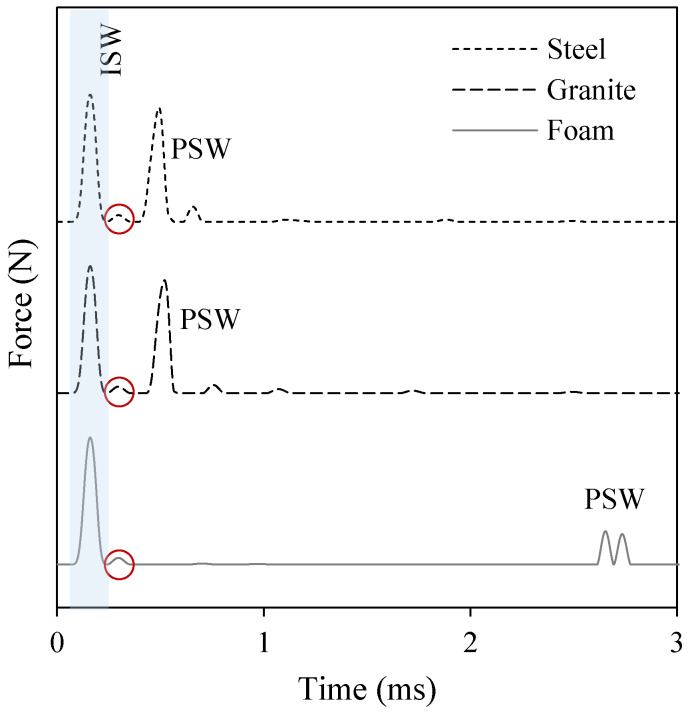
Solitary wave force profiles obtained from the numerical modeling of the interaction of solitary waves and steel, granite, and foam specimens.

Figure 15 shows the amplitude of the dynamic force measured at the sensor disk (site #5 in Figure 14a). The waveforms represent the average interaction force between the sensor particle and the adjacent particles (sites #4 and #6). As observed in the experiments (Figure 11a, Figure 12a or Figure 13a), Figure 15 shows that the ISW (highlighted in light blue background) is trailed by a small pulse emphasized with red circles. This pulse is the result of a rebound between the sensor disk and particle #6, caused by the local increase of contact stiffness between the disk and the sphere. The higher (with respect to the contact between two spheres) contact stiffness at the sensor particle results in the increase of its maximum particle velocity. As the ISW passes through the sensor and arrives at bead #6, the disk and the particle #6 separate. This yields a momentary zero interaction force at the sensor particle (Figure 15). Then, owing to its higher momentum, the sensor particle strikes particle #6 again, yielding to the small hump circled in Figure 15. The same mechanism causes the small pulses trailing the PSW in the steel and granite tests.

When the *transducer* was used above the foam board, both the experimental (Figure 12a) and the numerical (Figure 15) results show a twin PSW. Owing to its soft nature, the acoustic energy carried by the ISW causes a relatively large deformation chain/foam interface. This deformation induces separation between particles that are much larger than the separation that occurs when the chain is above stiff materials. When the reflected wave propagates back to the chain, the separation between bead #6 and the sensor particle and the separation of the sensor particle and bead #4 is large enough that two distinct impacts, i.e., peaks, are seen. The first peak is therefore the upward impact of particle #6 onto the sensor disk, and the second peak is the upward impact of the disk onto particle #4.

Figure 15 also confirms what was observed experimentally (Figure 11b, Figure 12b, or Figure 13b) about the ToF that is inversely proportional to the stiffness of the interface material. The results of the numerical study are summarized in Table 1. The numerical ToF predictions are in very good agreement with the experimental results displayed in Figure 11b, Figure 12b or Figure 13b. At most, the discrepancy between the numerical and the experimental values is within 2–3 data samples. As the thin foam board was placed on a rigid optical table, it is believed that the mechanical properties of foam only material were not representative of the real test object. As such, Young’s modulus foam listed in Table 1 was calibrated to match the numerical and the experimental results.

## 6. Discussion and Conclusions

In statistics, the Pearson’s correlation coefficient is generally used to measure the linear correlation between two variables. The coefficient can be comprised between +1 and −1, where 1 is the total positive linear correlation, 0 is no linear correlation, and −1 is the total negative linear correlation. Following past studies [59,60], Pearson’s correlation coefficient was used in the work presented in this article to measure the similarity between the waveforms collected with the PXI and the wireless platform. The values of the coefficients for the steel, foam, and granite were 0.975, 0.878, and 0.957, respectively. Although this observation needs data from more materials and a greater number of tests in order to be definitive, the trend of the coefficients seems to be related to Young’s modulus of the test specimen. In general, a coefficient greater than 0.8 is considered to be strong evidence of a linear relationship [61]. In this context, it means that it is very likely that the mean waveforms from the PXI and PCB are simply scaled versions of each other. The Pearson coefficients confirm the research hypothesis that the proposed wireless module can replace the wired configuration to perform NDE/SHM using HNSWs.

The quantitative results relative to the ToF are summarized in Table 2, which presents the average value of the ToF, and the corresponding standard deviation and coefficient of variation (CoV) for all the experiments conducted in this study. Additionally, the difference in percent between the numerical and the PCB-measured ToF is reported. Overall, it can be said that the experimental results are in very good agreement with the numerical values and the difference is within the margin associated with the sampling period of the instrumentation, i.e., related to the accuracy provided by the sampling frequency adopted in the experiments. By observing the standard deviations, they are within 1–2 data samples. In addition, the coefficient of variation is quite small proving the repeatability of the experiments.

In conclusion, in the study presented in this article, a wireless node was designed, assembled, and tested to support the generation, propagation, and detection of highly nonlinear solitary waves. The aim was to develop the components of a custom system that allows for structural health monitoring applications based on solitary waves. The development of the wireless sensor node included the design of a filter, a driver, and a mobile application. To prove the feasibility of the first prototype, three different interface materials were tested using two HNSW transducers. One transducer was conventionally wired to a National Instruments PXI running under LabView and one transducer was connected to a wireless node and driven with a mobile App using a tablet. The experimental results showed an excellent agreement with respect to each other and with respect to an analytical model that described the dynamic interaction between the waves and the material in dry contact with the chain. This is proof of the reliability of our wireless platform.

In the future, the wireless system needs to be improved over a few fronts. The sampling frequency should be increased to at least 1 or 2 MHz in order to reduce the sampling period and to make the system more sensitive to variations within the material being probed. The power necessary to drive the solenoid needs to be reduced in order to increase the battery life and allow the system to last longer before calling upon battery replacement. The development of a power solar cell to harvest energy would allow the wireless node to operate indefinitely. The latter would also be a suitable solution to power low voltage electronics. With the current design, the 3.7 V, 2500 mAh battery can power the low voltage electronics for 14 h. Besides harvesting energy with a solar module, another remedy could be adding both a separate smaller battery to power the Bluetooth module while it sleeps in a low power mode and hardware to allow the Bluetooth module to turn on the rest of the board once it receives a wake-up signal.

## Figures and Tables

**Table 1:** Mechanical properties, specimen thickness, and the predicted ToFs of the steel, granite, and foam specimens in the numerical study.

Material	Material Properties			Specimen Thickness (mm)	Numerical Time of Flight (ms)
	Density (kg/m^3^)	Modulus of Elasticity (GPa)	Poisson’s Ratio		
Steel	7800	200	0.3	6.35	0.333
Granite	2700	70	0.25	304.8	0.359
Foam	60	0.03	0.3	6.35	2.494

**Table 2:** Summary of the experimental and numerical results relative to the time-of-flight of the primary reflected solitary wave. The CoV represents the coefficient of variation, i.e., the ration of the standard deviation to the mean value.

Parameter	Material		
	Steel	Granite	Foam
ToF numerical (μsec)	333	359	2494
ToF experim.: PCB mean (μsec)	320	347	2510
ToF experim.: PCB Standard deviation (μsec)	8.20	12.1	20.5
ToF experim.: PCB CoV (%)	2.56	3.47	0.82
ToF experim.: PXI mean (μsec)	313	341	2551
ToF experim.: PXI Standard deviation (μsec)	8.76	14.2	27.1
ToF experim.: PXI CoV (%)	2.79	4.16	1.06
Numerical/PCB difference (%)	3.90	3.34	0.64
